# The CAD-score web server: contact area-based comparison of structures and interfaces of proteins, nucleic acids and their complexes

**DOI:** 10.1093/nar/gku294

**Published:** 2014-05-16

**Authors:** Kliment Olechnovič, Česlovas Venclovas

**Affiliations:** 1 Institute of Biotechnology, Vilnius University, Graičiūno 8, Vilnius LT-02241, Lithuania; 2 Faculty of Mathematics and Informatics, Vilnius University, Naugarduko 24, Vilnius LT-03225, Lithuania

## Abstract

The Contact Area Difference score (CAD-score) web server provides a universal framework to compute and analyze discrepancies between different 3D structures of the same biological macromolecule or complex. The server accepts both single-subunit and multi-subunit structures and can handle all the major types of macromolecules (proteins, RNA, DNA and their complexes). It can perform numerical comparison of both structures and interfaces. In addition to entire structures and interfaces, the server can assess user-defined subsets. The CAD-score server performs both global and local numerical evaluations of structural differences between structures or interfaces. The results can be explored interactively using sortable tables of global scores, profiles of local errors, superimposed contact maps and 3D structure visualization. The web server could be used for tasks such as comparison of models with the native (reference) structure, comparison of X-ray structures of the same macromolecule obtained in different states (e.g. with and without a bound ligand), analysis of nuclear magnetic resonance (NMR) structural ensemble or structures obtained in the course of molecular dynamics simulation. The web server is freely accessible at: http://www.ibt.lt/bioinformatics/cad-score.

## INTRODUCTION

Quantitative comparison of two structures of the same biological macromolecule or complex is a very common but by no means trivial task. One such example is the comparison of a model with the experimentally determined (reference) structure. This is a crucial element in the evaluation of computational methods for protein or RNA 3D structure prediction and for protein–protein or protein–DNA (RNA) docking. The analysis of molecular dynamics simulation results involves comparison of conformational changes within the structure at different time points. Another common example is the comparison of protein or nucleic acid crystal structures solved either using different conditions or in different states (e.g. free protein and the same protein in complex with DNA, protein with and without a bound ligand).

Many commonly used structure comparison methods are based on measurements of distance between equivalent atoms after superposition of structures as rigid bodies. The Root-Mean-Square deviation (RMSD) due to its universal nature is one of the most popular methods of this class. RMSD can be computed on a selected set of atoms for any type of structure. However, RMSD values are meaningful only when the structural differences are small and fairly equally distributed. RMSD is overly sensitive to large local deviations so that even one or two outlying residues may give a misleading impression about the overall structural similarity. In attempt to overcome drawbacks of RMSD, a number of alternative superposition-based scores have been introduced. Some of the best-known alternatives for proteins include Global Distance Test Total Score (GDT-TS) (1,2) and TM-score (3). Both can be computed using the corresponding web servers (3,4). Structure-superposition-based scores developed specifically for RNA include deformation index (5), deformation profile (5), and RNAlyzer (6), the latter method implemented as a dedicated web server. Although common, rigid structure superposition-based methods typically fail to account for the biologically relevant flexibility such as movements of protein loops or domains.

Another class of methods is based on comparison of distances or interactions within one structure with the corresponding distances or interactions within the other structure. These methods have the advantage of being superposition-independent. Examples of such methods include Local Distance Difference Test (7) for proteins (available as a web server) and Interaction Network Fidelity (5) for RNA. Even though superposition-independent methods avoid superposition-related problems, there are many ways of how to represent structural similarity and the open question is which one is the best. In this regard, the concept of contact area (8) is an interesting way to represent not only physical residue–residue interactions but also their strength. Using this concept we have recently developed Contact Area Difference score (CAD-score), a method to quantify both local and global similarity of structures and interfaces (9). CAD-score was initially developed for proteins; however, most recently we extended its application to RNA 3D structure (10). In general, the universal nature of the method makes it applicable to any major type of macromolecular structures. Here, we describe a web-based interface for the CAD-score computation and interactive analysis of the results for proteins, nucleic acids and their complexes. The CAD-score web server is free and open to all and there is no login requirement.

## MATERIALS AND METHODS

### Interatomic contacts

**Figure 1.**
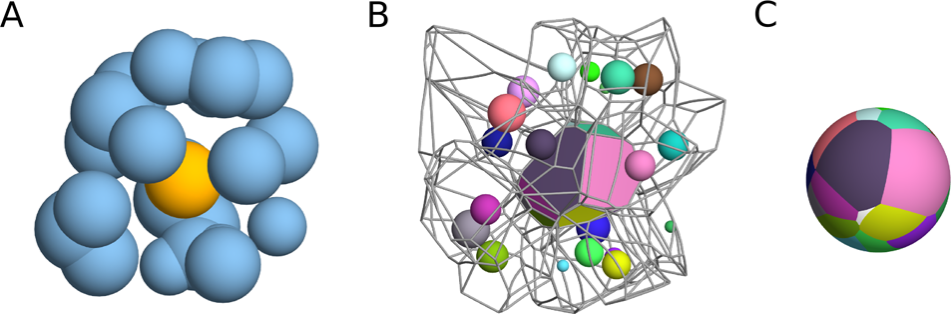
Illustration of the derivation of interatomic contacts. (**A**) The considered atom (yellow) surrounded by neighboring atoms (blue). (**B**) Voronoi cells of the atoms from (A). The small colored spheres correspond to the neighboring atoms. The Voronoi cell of the considered atom has faces colored according to the colors of the neighbors. The Voronoi cells of the neighbors are displayed as wire frames. (**C**) The contact sphere of the considered atom, subdivided into contact areas according to the Voronoi tessellation.

Interatomic contacts within the input molecular structure are derived from the Voronoi tessellation of 3D balls, where balls correspond to heavy atoms of van der Waals radii (11). Two atoms are considered to be in contact if they are neighbors in the Voronoi tessellation and the water molecule cannot fit between them. For each atom a contact sphere is defined, which is a sphere of the radius equal to the sum of van der Waals radius of the atom and the standard radius of the water molecule (1.4 Å). The entire surface of the contact sphere is partitioned into either interatomic contact areas or solvent accessible areas according to the Voronoi tessellation. Figure 1, generated using Voroprot (12), illustrates how interatomic contacts are derived.

### Contacts between residues

**Figure 2.**
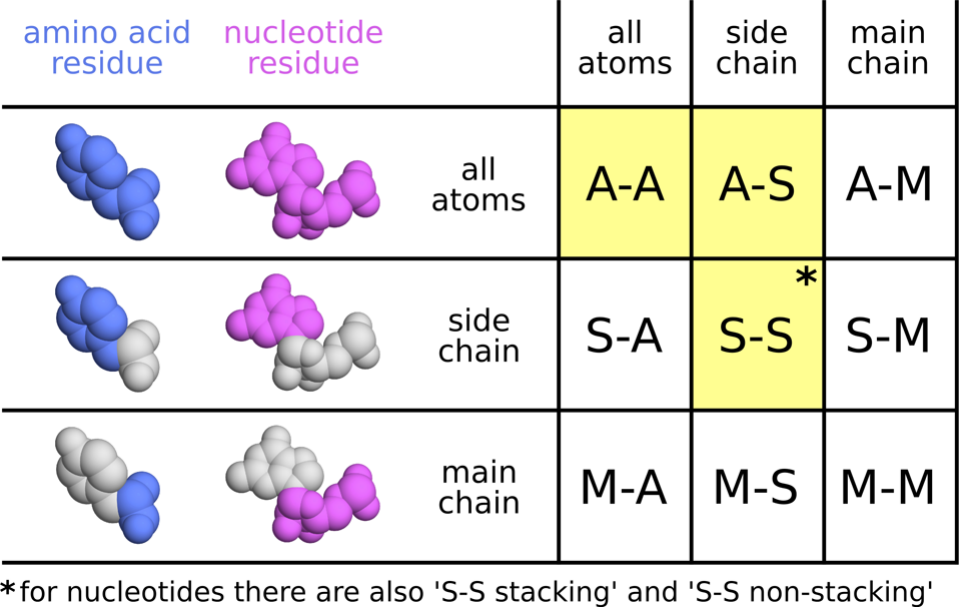
CAD-score variants based on standard subsets of residue (amino acid or nucleotide) atoms. ‘A’, ‘S’ and ‘M’ denote all atoms, side chain (base) and main chain (backbone), respectively. CAD-score variants highlighted in yellow are provided as part of the web server results that can be explored interactively.

Residues are considered to be in contact if they have at least one interatomic contact. Contact areas between residues are derived by simply grouping interatomic contact areas. Interatomic contacts that correspond to covalent bonds between adjacent residues are not considered. In addition to contacts between entire residues, contacts between subsets of residue atoms can be defined. Two standard subsets are considered: main chain and side chain for proteins and correspondingly backbone and base for nucleic acids. The resulting CAD-score variants are shown in Figure 2. All CAD-score variants are computed but for the interactive analysis the web server provides only five CAD-score variants (‘A-A’, ‘A-S’, ‘S-S’, ‘S-S stacking’ and ‘S-S non-stacking’) deemed to be most useful. The last two variants are applicable only for nucleic acids. If desired, all CAD-score variants can be downloaded as a text table.

### Scoring structural similarity

Structural similarity is quantified based on how closely corresponding contact areas in the two structures agree. Let us denote the contact between residues *i* and *j* in the reference structure (target) as (*i*,*j*), their contact area as *T_(i,j)_*, and the contact area between the corresponding residues in the structure to be compared (model) as *M_(i,j)_*. If *i* and *j* are not in contact in the model, then *M_(i,j)_* = 0. Let us denote the set of all the residue–residue contacts in the target structure as *G*. Then, the similarity score between the target and the model is defined as follows:

(1) \begin{equation*}
{\rm CAD-score} = 1 - \frac{{\sum\nolimits_{(i,j) \in G} {{\rm min}(|T_{(i,j)} - M_{(i,j)} |,T_{(i,j)} )} }}{{\sum\nolimits_{(i,j) \in G} {T_{(i,j)} } }}
\end{equation*}

CAD-score values are always within the [0,1] range with CAD-score = 1 meaning identical contacts.

In addition to the global score (Equation 1), for the analysis and visualization of local differences between the two structures two types of local error values (raw and normalized) are derived for every residue. A raw local error for residue *i* is defined as follows:

(2) \begin{equation*}
\delta (i) = \sum\nolimits_{(i,j) \in G} {\min (|T_{(i,j)} - M_{(i,j)} |,T_{(i,j)} )}
\end{equation*}

A normalized local error, which is referred later in the text simply as ‘local error’, is a raw local error divided by the sum of the corresponding target contact areas:

(3) \begin{equation*}
\varepsilon (i) = \frac{{\delta (i)}}{{\sum\nolimits_{(i,j) \in G} {T_{(i,j)} } }}
\end{equation*}

Local error values for individual residues may show large variation. To make the signal less noisy, both raw and normalized local errors can be smoothed along the residue sequence using a window of *w* residues to the left and to the right:

(4) \begin{equation*}
\delta _w (i) = \frac{{\sum\nolimits_{k \in [ - w,w]} {\delta (i + k)} }}{{2w + 1}}
\end{equation*}

(5) \begin{equation*}
\varepsilon _w (i) = \frac{{\sum\nolimits_{k \in [ - w,w]} {\delta (i + k)} }}{{\sum\nolimits_{k \in [ - w,w]} {\sum\nolimits_{(i+k,j) \in G} {T_{(i + k,j)} } } }}
\end{equation*}

## CAD-SCORE WEB SERVER DESCRIPTION

### Input

#### Input data

The inputs to the web server are macromolecular structure files in Protein Data Bank (PDB) format: one file for the reference structure (target) and one or more files of structures (models) to be compared with the target. A user is also asked to specify the type of the input structures: proteins, nucleic acids or protein–nucleic acid complexes.

By default, the web server verifies that the residue sequence, residue numbering and chain naming in each model are consistent with the target structure. If inconsistencies are detected, the web server stops and reports an error. The user can alter this behavior by selecting an option to allow mismatches between model and target sequences. In such case only the consistency of residue numbering and chain naming is verified. This option might be useful for comparison of structures with one or few mismatches such as native structures and point mutants.

#### Evaluation modes

The CAD-score web server provides a flexible way to choose which residue–residue contacts to analyze. The most straightforward choice is to analyze contacts within the entire structure.

Another option is to evaluate inter-chain interfaces. In this case by default only contacts between residues belonging to different chains are analyzed. The user may choose to extend the reference set of contacts by additionally including contacts between the interface residues from the same chain. In both cases the reference set of interface residues is the same, only the contact reference sets differ.

Finally, the most flexible option is to instruct the CAD-score web server to analyze only contacts between custom selections of residue groups. A selection can be specified by writing chain names or residue identifiers (or ranges of residue identifiers) in a simple notation. Examples of custom selections are given in Table 1.

**Table 1.** Examples of custom selection strings that define subsets of residue–residue contacts

String	Meaning	Examples of evaluation target
(A)(B)	Contacts between chains A and B	Interface between two subunits in a multi-subunit structure
(A,B)(C)	Contacts of chains A and B with C	Interface between a protein dimer and the RNA in a protein–RNA complex
(A)(A)	Contacts between residues within chain A	Contacts within a single subunit in a multi-subunit structure
(A1-A9,A21-A90)(B1-B90)	Contacts between two explicitly specified groups of residues	Interface between two domains in a multi-subunit structure

### Output

#### Representation of global scores

**Figure 3.**
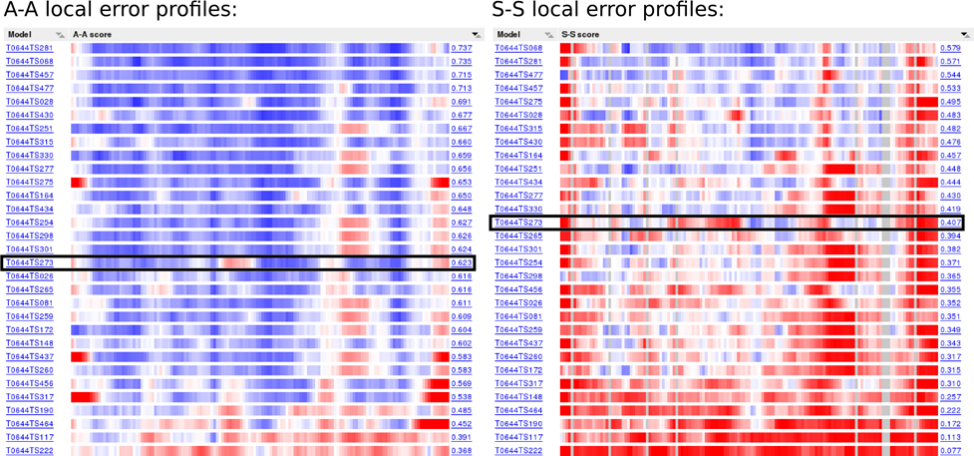
Example of a global view of A-A and S-S local error profiles generated for some models of the CASP10 (www.predictioncenter.org/casp10) prediction target T0644. Black frames indicate profiles for the same model, TS273.

The default view of the results generated upon the completion of a user-submitted job is a summary, presented as a sortable table of global score values. Independently of the molecule type the table has columns for ‘A-A’, ‘A-S’ and ‘S-S’ CAD-scores. Other table columns are specific for the macromolecule type. In the case of proteins, TM-score, GDT-TS and GDT-HA scores as computed by the TM-score software (3) are included. In the case of nucleic acids, ‘S-S’ CAD-score evaluating base–base contacts is further subdivided into S-S stacking and S-S non-stacking scores. A table of global scores for protein–nucleic acid complexes has only the columns that are available for both proteins and nucleic acids. A table of global scores including all CAD-score variants is also available for downloading in the flat text format. In addition to the summary table, there are sortable tables of global scores for specific CAD-score variants for all processed models. A global score for each model in these tables is accompanied by the color-coded profile of local errors (described in detail in the following section). This view is particularly useful for the simultaneous analysis of multiple models as it enables to contrast and compare local discrepancies of individual models in the overall context (Figure 3).

#### Visualization of local errors

Each global CAD-score value in either sortable table is linked to a detailed report of local errors for the corresponding model. Local errors are primarily displayed as profile images where the value of the local error for each analyzed residue is color-coded using blue–white–red gradient. Blue and red colors represent good and poor agreement, respectively. For each CAD-score variant four profile versions are generated using smoothing windows of 0 (no smoothing), 1, 2 and 3 residues on both sides of each analyzed residue. Additionally, four profiles of raw local errors with the same smoothing windows are generated for comparison. The actual values of both raw and normalized local errors without smoothing can be viewed in plain text format. The combined contacts file detailing corresponding contact areas for each residue in the target and the model is also available as a text file for the off-line analysis.

**Figure 4.**
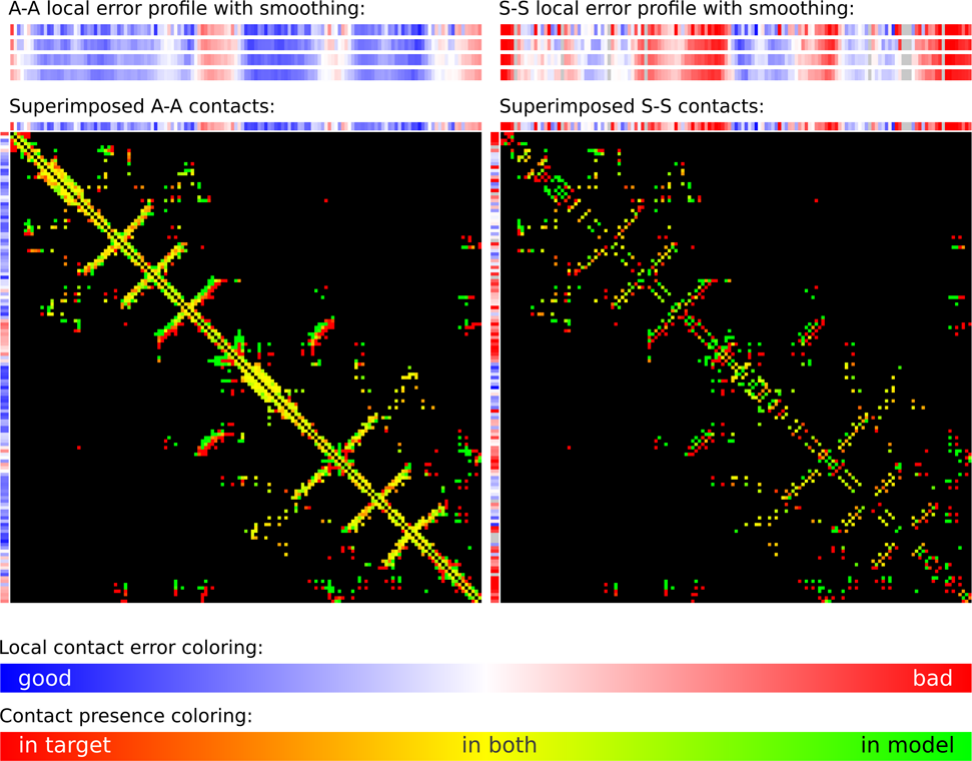
Local error profiles and superimposed contact maps of A-A and S-S contacts for the model highlighted in Figure 3.

**Figure 5.**
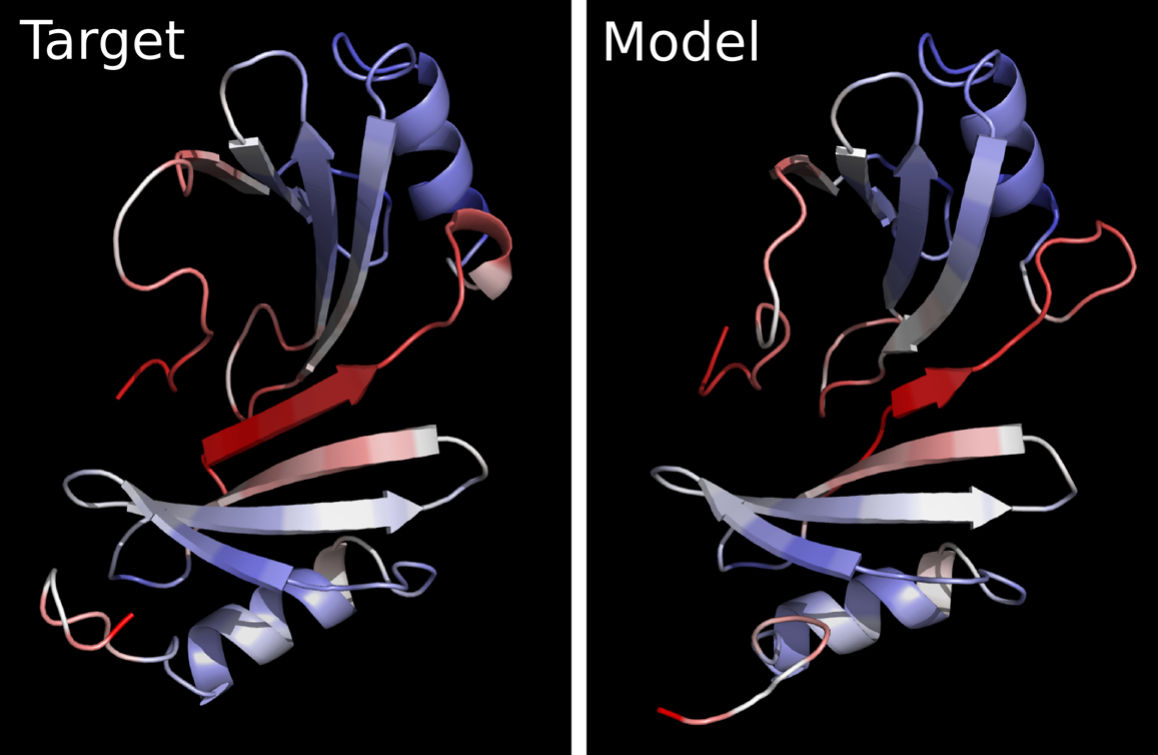
Experimentally solved (target) and predicted (model) structures colored according to the local errors in the model highlighted in Figure 3.

**Figure 6.**
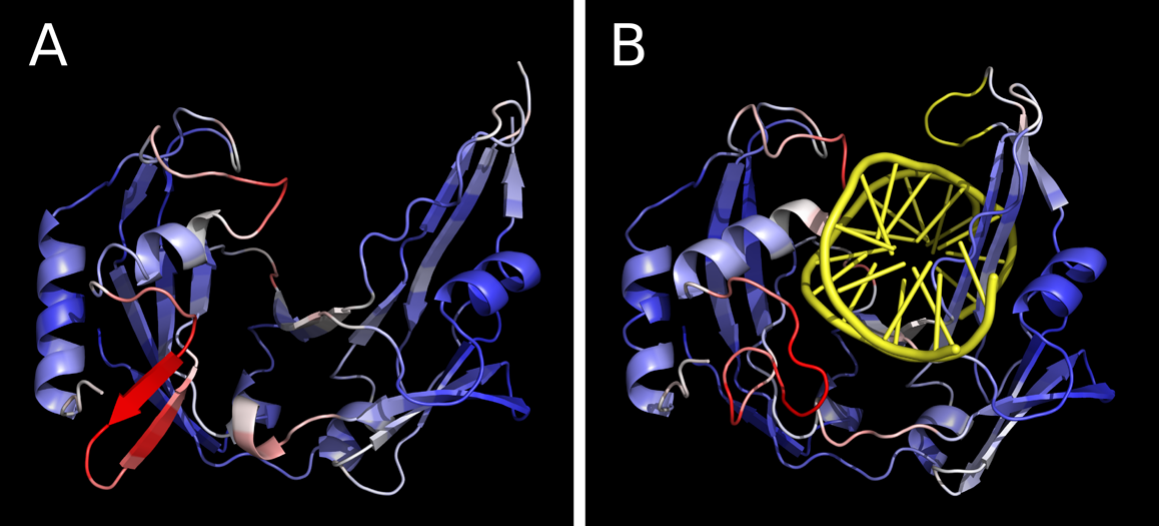
Local differences between the structures of the restriction endonuclease BcnI crystallized (**A**) in the apo form (PDB id: 2odh) and (**B**) in complex with the cognitive DNA (PDB id: 2q10). The DNA and the loop unresolved in the apo form are shown in yellow.

Figure 4 provides examples of local error profiles and their relation to the superimposed contact maps for the model and the target. Such maps, generated by the server, show contacts represented as colored points on the black background. Residue contacts in the target are red, in the model are green, and the color of coinciding contacts consists of red and green components mixed with a ratio proportional to the corresponding contact areas in the target and in the model. Therefore, yellow color indicates that the areas of corresponding contacts are of approximately same size. Images of both local error profiles and contact maps are interactive: a user can click on them to see the corresponding residue numbers. Another way to analyze local errors is to visualize them in the context of 3D structures with Jmol, an interactive molecular viewer. Local errors are converted into the B-factor values of PDB files for both target and model and are represented by the same color gradient as in the corresponding linear profiles. Local discrepancies mapped onto 3D structures are exemplified with the predicted and experimental protein structures (Figure 5) and with the two X-ray structures of a protein crystallized in a free state and with the bound DNA (Figure 6).

**Figure 7.**
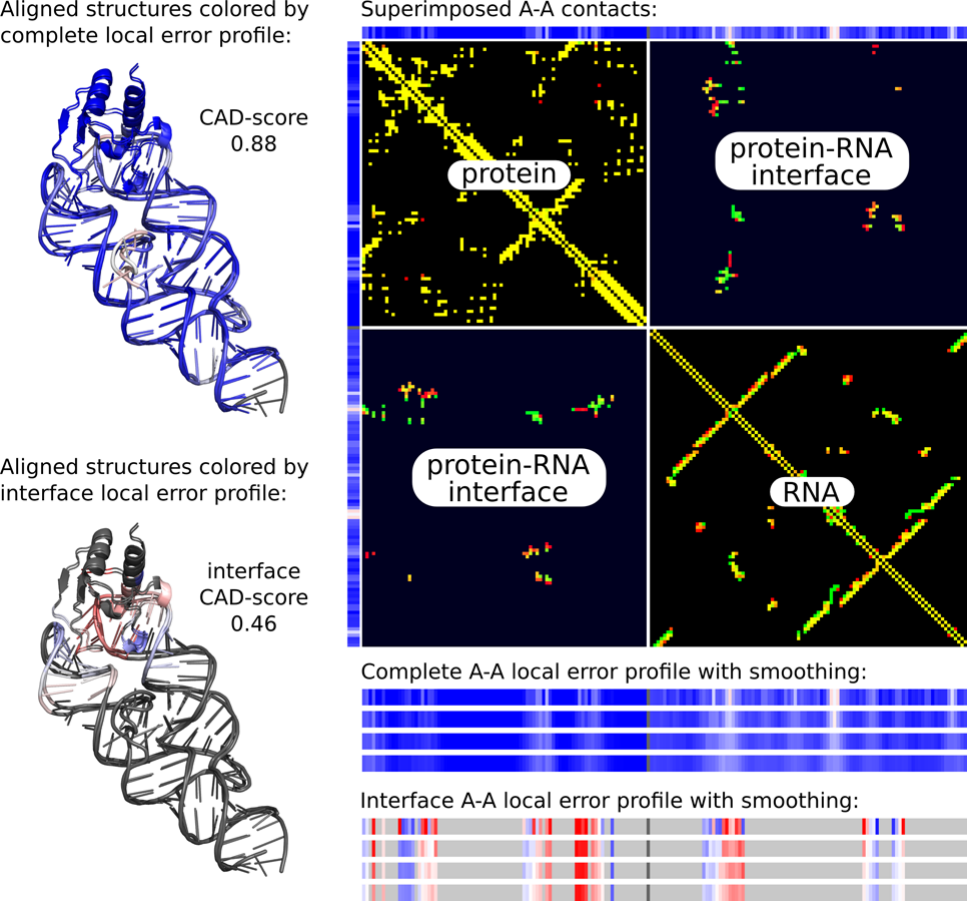
Example of the evaluation of a protein–RNA complex model against the reference structure. The model corresponds to the structure 1364 from the decoy set used in the assessment of protein–RNA docking solutions.

Local error profiles can also be used when analyzing subsets of residue–residue contacts. An example in Figure 7 features comparison of a decoy and the native structure of a protein–RNA complex used in a study aimed at scoring protein–RNA docking solutions (13). The complete local error profile shows that in the decoy structure the contacts both inside the protein chain and inside the RNA chain are reproduced relatively accurately. However, the comparative contact map of the protein–RNA complex shows that contacts between protein and RNA differ significantly. Therefore, it is also useful to analyze the local error profile produced only for the interface residues.

## CONCLUSION

The web server provides a simple and intuitive interface for the use of the CAD-score method in the interactive manner. In particular, the server features highly interactive visualization options of local contact differences. The server is universal in several ways. It accepts both single-chain and multi-chain structures, works with all the major types of macromolecules (proteins, RNA, DNA and various complexes), allows flexible designation of substructures for the analysis and performs both global and local evaluation of structural differences. Thus, the CAD-score server provides a single framework for addressing a variety of questions related to structural similarity for all the major types of biological macromolecules.

## FUNDING

Research Council of Lithuania [PRO-02/2012]; European Social Fund under the Global Grant measure [VP1-3.1-ŠMM-07-K-03-004].

*Conflict of interest statement*. None declared.
